# An Unusual Case of 5-Oxoproline Acidosis

**DOI:** 10.1016/j.xkme.2025.101236

**Published:** 2025-12-29

**Authors:** Nils Müller, Anne Krüger, Alexander Reshetnik

**Affiliations:** Charité – Universitätsmedizin Berlin, corporate member of Freie Universität Berlin and Humboldt-Universität zu Berlin, Medizinische Klinik mit Schwerpunkt Nephrologie und Internistische Intensivmedizin, Berlin, Germany

**Keywords:** 5-oxoprolin, flucloxacillin, high anion gap, oxacillin, metabolic acidosis, pyroglutamic

## Abstract

Although metabolic acidosis is a common phenomenon, its differential diagnoses include a variety of rare conditions. Among the causes of high anion gap metabolic acidosis is an accumulation of 5-oxoproline or pyroglutamic acid. It is commonly understood that development can occur in the context of high-dose paracetamol intake and either relevant clinical risk factors or comedication with flucloxacillin or oxacillin. As a rare condition, it has become well-known only to specialized clinicians. We reported a case of high anion gap metabolic acidosis caused by 5-oxoproline in a middle-aged male treated only with high-dose flucloxacillin. Our patient had neither a history of paracetamol use nor classical risk factors to a degree, making the development of 5-oxoproline/pyroglutamic acidosis plausible. Although there have been plenty of case reports of this condition because of the coadministration of paracetamol/acetaminophen and flucloxacillin/oxacillin, to our knowledge, this is only the second published case of 5-oxoproline acidosis without paracetamol use, making it either highly unusual or potentially underdiagnosed. Therefore, we suggest considering 5-oxoproline/pyroglutamic acidosis when evaluating the etiology of metabolic acidosis in patients on high-dose flucloxacillin/oxacillin, even without a history of concomitant or previous paracetamol exposure or other classical risk factors.

## Case presentation

A 56-year-old male who had undergone coronary arterial bypass surgery 1^1/2^ months prior presented to the emergency room with brown-putrid secretion from the sternal wound for 1 day, a mild cough for 3 days, and a positive severe acute respiratory syndrome coronavirus 2 (SARS-CoV-2) rapid antigen test. Inflammatory marker levels were markedly elevated. A chest computed tomography scan confirmed anterior mediastinitis and sternal osteomyelitis. Blood cultures were drawn, and broad-spectrum antibiotic therapy was initiated using piperacillin/tazobactam and vancomycin. A polymerase chain reaction confirmed SARS-CoV-2 infection, but besides the mild cough, there were no other clinical symptoms of coronavirus disease 2019.

The day after admission, the patient underwent surgery for his deep sternal wound infection and osteomyelitis. The wound was managed from here forth with vacuum dressings. The cultures showed growth of an oxacillin-sensitive strain of *Staphylococcus aureus* in blood cultures (4 out of 4 before therapy, 2 out of 4 the day after therapy was initiated) and in both the superficial wound swab and the intraoperative tissue sample. Hence, antibiotic therapy was changed to intravenous flucloxacillin 2 g 6 times daily. Under therapy, the patient showed clinical improvement, and inflammatory marker levels decreased. Pain was managed using metamizole and opioids. Six days after admission, he was transferred to an associated hospital for further management. His therapy was continued without relevant changes before being transferred back to plastic surgery in our hospital. Meanwhile, SARS-CoV-2 polymerase chain reactions had turned negative, and the patient still had no related symptoms. Ten days after admission, blood gas analysis showed a newly developed metabolic acidosis (pH 7.29, HCO_3_^−^ concentration 10.7 mmol/L [ABE -14.4], venous pCO_2_ 23 mmHg, lactate level 7 mg/dL [< 16 mg/dL]). His kidney function was mildly impaired with an estimated glomerular filtration rate (CKD-EPI creatinine) around 45 mL/min/1.73 m^2^ from a baseline of 90 mL/min/1.73 m^2^. Nephrology was consulted because of profound metabolic acidosis. Further analysis showed a high anion gap of 17 mmol/L (normal range 7 ± 4 mmol/L).^1^ Urine analysis revealed a pH of 5.5, no glucosuria, mild proteinuria (protein-creatinine ratio of 500 mg/g), and no relevant albuminuria (albumin-creatinine ratio of 45 mg/g). There was no history of relevant paracetamol usage. The extent of renal failure did not explain the profound metabolic acidosis. After excluding the usual causes of metabolic acidosis with high anion gap, we checked for rare causes, sent urine for 5-oxoproline concentration, and switched antibiotic therapy from flucloxacillin to cefazolin while awaiting results. Acidosis was treated by large amounts of intravenous infusions of bicarbonate and did not recur afterward. At the same time, because of severe septic shock secondary to wound site infection with *Serratia marcescens* bacteriaemia, the patient briefly developed anuric, then oliguric kidney injury and required continuous renal replacement therapy. After recovery from sepsis and acute kidney injury, his estimated glomerular filtration rate remained at 45 mL/min/1.73 m^2^. Vacuum dressing therapy for his sternal wound was continued, and he was discharged home after a total of 2 months of treatment.

## Results

The urine sample revealed a 5-oxoproline concentration in the urine of 533 mg/g creatinine (normal value < 60 mg/g creatinine). A follow-up sample 5 days after discontinuation of flucloxacillin showed an almost normalized value (89.9 mg/g creatinine). The onset of acidosis was rather sudden on day 8 of treatment with flucloxacillin and day 10 after admission. Rigorous study of the medical record identified 3 separate times when the patient had received a single shot of 1 g of paracetamol intravenously: once on the day of admission and thus 2 days before receiving flucloxacillin (10 days before the onset of acidosis), and twice after the acidosis had already developed. None of them correlated with the onset or worsening of the acidosis. After the patient recovered, both he and his wife denied any paracetamol usage at home before admission. He reported daily beer consumption (300-1,000 mL) and eating a Central European diet including vegetables, dairy, eggs, and meat. Neither imaging nor laboratory values showed any signs of liver disease. The patient denied having had issues with kidneys, liver, electrolytes, or acid-base homeostasis previously, nor did he have any knowledge of any of these among his relatives.

## Discussion

**Figure. 1:**
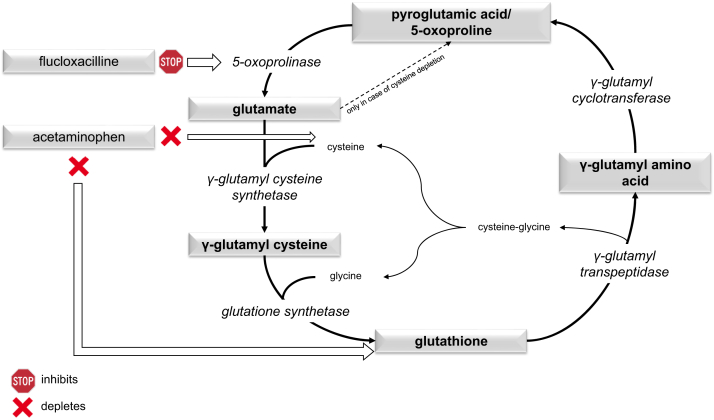
Pathophysiology of 5-oxoproline acidosis.

Metabolic acidosis caused by 5-oxoproline is a known rare metabolic disorder in which the γ-glutamyl cycle involved in amino acid catabolism is impaired, leading to an accumulation of 5-oxoproline. It is generally accepted that oxoproline acidosis can develop when certain factors combine to inhibit this cycle at different points or make people susceptible to glutathione or cysteine level depletion (Fig 1). The most commonly cited risk factors are female sex, severe or prolonged sepsis, advanced liver disease, pronounced malnutrition, usually in combination with prolonged use of high doses of paracetamol. Especially, paracetamol is present in nearly all reported cases. It is noted that the combination of paracetamol and flucloxacillin, oxacillin, or dicloxacillin sharply increases the risk for the development of acidosis so that nonpharmaceutical risk factors are less relevant then. This led to a warning of this combination from the European Medicines Agency’s Pharmacovigilance Risk Assessment Committee in October 2017, which has since been included in the flucloxacillin package leaflet.^2^

A review of the literature identified 79 publications with case reports about 5-oxoproline-associated acidosis that involved flucloxacillin, oxacillin, or dicloxacillin. Among them, all but 2 of the reported cases were because of concomitant use with paracetamol. Among the 2 cases without paracetamol, 1 included the usage of other antibiotics inhibiting oxoproline degradation (ciprofloxacin and netilmicin).^3^ When specified, paracetamol was used at high doses over an extended period, thus continuously inhibiting glutathione metabolism. Other risk factors varied. Therefore, paracetamol appears to be a key element in developing this condition. Because of its toxic metabolite N-acetylbenzoquinonimine, which irreversibly binds glutathione, it depletes glutathione stores. The rate-limiting factor in the restoration of glutathione stores is the addition of glycine to γ-glutamylcysteine mediated by glutathione synthetase, resulting in some of it becoming a substrate for γ-glutamyl-cyclotransferase and thus being converted to 5-oxoproline. Because flucloxacillin inhibits the enzyme responsible for the breakdown of 5-oxoproline, it accumulates and acidosis develops. (Fig 1).^4^ For 5-oxoproline acidosis to develop without flucloxacillin, it appears that an ATP-depleting cycle can develop. For this to occur, cysteine depletion is required, which can be caused by paracetamol. In this situation, glutamate gets phosphorylated as usual, but because of the missing cysteine, it autocyclizes to 5-oxoproline again. To degrade 5-oxoproline to glutamate, ATP is required by the 5-oxoprolinase. This futile circle depletes ATP stores in the cell. Once low enough, the 5-oxoprolinase ceases to function normally, and severe accumulation develops (Fig 1).^5^^,^^6^

We reported a case of metabolic acidosis caused by 5-oxoproline without paracetamol/acetaminophen. We could find only one previous report where 5-oxoproline acidosis developed without known paracetamol use: a 64-year-old multimorbid woman who was admitted to hospital with an *S. aureus* prosthetic joint infection and treated with high-dose flucloxacillin. Her pre-existing conditions, many of which were risk factors for the development of 5-oxoproline acidosis, included chronic pain syndrome, chronic hyponatremia, systemic lupus erythematosus, rheumatoid arthritis, adrenal insufficiency, a seizure disorder, and Crohn’s disease. Her body mass index was 21 kg/m^2^. Although it is reported that this patient’s “medication list” did not include paracetamol or other “drugs with metabolic acidosis listed as adverse events,” there is no mention of whether the patient may have used self-administered paracetamol as an over-the-counter medication for her chronic pain.^7^

To our knowledge, our case is the second report of 5-oxoproline acidosis following high-dose flucloxacillin without paracetamol use. Our patient had several factors that could predispose him to glutathione depletion, such as chronic inflammation (osteomyelitis) and low-grade malnutrition because of chronic low-dose alcohol consumption and chronic pancreatitis. However, the latter did not prevent him from being of normal body weight and gaining body weight without enzyme replacement therapy after his initial coronary bypass surgery. We do not believe that his mildly symptomatic SARS-CoV-2 infection played a relevant role. A review of the literature found no data suggesting an association between SARS-CoV-2 infection and glutathione depletion or 5-oxoproline-associated acidosis.

In comparison to our case, the patient in the previously reported case appears to be more severely ill, with an extensive medical history significantly increasing the risk of glutathione depletion, while our patient had weight gain before hospital admission and the development of metabolic acidosis. We were also able to rule out self-medication with over-the-counter paracetamol.

Although several risk factors were present in our patient, it still seems highly unusual for him to have developed 5-oxoproline acidosis. Whether there is an underlying genetic susceptibility to explain why this condition is not more common remains speculative.

In conclusion, our case report extends the evidence that high-dose flucloxacillin alone is capable of inducing 5-oxoproline metabolic acidosis without the involvement of other substances that inhibit glutathione metabolism. Therefore, 5-oxoproline acidosis should also be considered when evaluating the etiology of metabolic acidosis in patients on high-dose flucloxacillin, even without a history of concomitant or previous paracetamol exposure.
